# The perception of time while perceiving dynamic emotional faces

**DOI:** 10.3389/fpsyg.2015.01248

**Published:** 2015-08-21

**Authors:** Wang On Li, Kenneth S. L. Yuen

**Affiliations:** ^1^Department of Counselling and Psychology, Hong Kong Shue Yan UniversityBraemar Hill, Hong Kong, China; ^2^Focus Program Translational Neuroscience, Neuroimaging Center Mainz, Johannes Gutenberg University Medical Center MainzMainz, Germany

**Keywords:** emotion, face perception, time perception, approach avoidance, dynamic emotional faces

## Abstract

Emotion plays an essential role in the perception of time such that time is perceived to “fly” when events are enjoyable, while unenjoyable moments are perceived to “drag.” Previous studies have reported a *time-drag effect* when participants are presented with emotional facial expressions, regardless of the emotion presented. This effect can hardly be explained by induced emotion given the heterogeneous nature of emotional expressions. We conducted two experiments (*n* = 44 and *n* = 39) to examine the cognitive mechanism underlying this effect by presenting dynamic sequences of emotional expressions to participants. Each sequence started with a particular expression, then morphed to another. The presentation of dynamic facial expressions allows a comparison between the time-drag effect of homogeneous pairs of emotional expressions sharing similar valence and arousal to heterogeneous pairs. Sequences of seven durations (400, 600, 800, 1000, 1200, 1400, 1600 ms) were presented to participants, who were asked to judge whether the sequences were closer to 400 or 1600 ms in a two-alternative forced choice task. The data were then collated according to conditions and fit into cumulative Gaussian curves to estimate the point of subjective equivalence indicating the perceived duration of 1000 ms. Consistent with previous reports, a feeling of “time dragging” is induced regardless of the sequence presented, such that 1000 ms is perceived to be longer than 1000 ms. In addition, dynamic facial expressions exert a greater effect on perceived time drag than static expressions. The effect is most prominent when the dynamics involve an angry face or a change in valence. The significance of this sensitivity is discussed in terms of emotion perception and its evolutionary significance for our attention mechanism.

## Introduction

Factors affecting the perception of time are well-documented. Following the pioneering work of François (1927) and Hoagland (1933), the influence of various internal and external contexts on the perception of time became one of the major focuses of cognitive psychology. “Time flying” and “time dragging” have been demonstrated and investigated in various studies involving both adults and children (Droit-Volet and Wearden, 2002; Droit-Volet et al., 2004a,b; Droit-Volet and Rattat, 2007; Gil et al., 2007; Gil and Droit-Volet, 2011; Grommet et al., 2011). Emotion has been found to play an essential role in the aforementioned phenomena. In particular, it is commonly found that when one enjoys an event, one perceives the passage of time faster than the actual duration, or “time flying” (Agarwal and Karahanna, 2000). In contrast, when one experiences an event as distressing or boring, they perceive the passage of time as longer than the actual duration and a feeling of “time dragging” is experienced.

The number of events in a given period is found to be positively correlated with the perceived passing of time (Gibbon et al., 1984; Burle and Casini, 2001; Wearden, 2004; Ulrich et al., 2006). Events are registered mentally as “ticks” and perceived to occupy time. The time occupied is not absolute but relative to the number of “ticks.” As such, a greater number of events happening in a given time duration will lead to an expansion of perceived time. The internal-clock model (Treisman, 1963) conceptualized this mechanism, and was later developed into scalar timing theory (Gibbon et al., 1984, 1997). The latter proposed that the internal clock is composed of three components: a pacemaker, a switch, and an accumulator. The pacemaker is a pulse generator; its function is to generate time-pulses and send them to the accumulator through the switch. The switch serves as a pulse guard that signals the start and the end of the timed event. The pulses are gathered by the accumulator and then converted into perceived duration. This mental model is widely successful in formulating testable hypotheses for time perception (Buhusi and Meck, 2005). For example, physical stimulation is proven to be sufficient in perturbing this mental process and to influence the perception of time (Treisman et al., 1994).

Recent studies have suggested that both arousal and attention can influence the internal clock (Gil and Droit-Volet, 2011). Increasing the level of arousal appears to increase the pulse-generating speed of the pacemaker. As a result, event duration is filled with more pulses and perceived as longer, resulting in “time dragging” (for a review, see Droit-Volet and Meck, 2007). Alternatively, attention influences one's perception of time by affecting the function of the switch. According to Zakay and Block (1996), the functioning of the switch requires attention resources. When attention is distracted from time perception, the switch cannot function properly. Time-pulses are then lost and ultimately time is perceived as shorter since less time-pulses are accumulated. To conclude, arousal influences time perception by changing the pulse-generating speed of the pacemaker, whereas attention affects time perception by moderating the function of the switch (Zakay and Block, 1997).

Induced emotion is also found to influence the perception of time. The individual's emotional state acts as a prime that prospectively affects time judgment on subsequent stimulus presentation. Since emotion can both provoke one's arousal and distract one's attention, the findings on the role of emotion in perception of time in humans were mixed (Lui et al., 2011). While some researchers have found a *time-drag effect* of emotion (Droit-Volet et al., 2004a), other found a completely opposite effect (Lui et al., 2011). The picture is even more complex as different emotions have been found to exert different effects on time perception. Gil and Droit-Volet (2011) found that anger, fear, joy, and sadness produce perceptions of “time dragging,” whereas shame produces perceptions of “time flying,” and disgust has no effect on time perception. They explained that different emotions with different levels of valence and arousal induce differential approach-avoidance motivations. For instance, emotions inducing salient avoidance enhance attentional resources allocated to detecting potential threats in the surrounding environment. The enhanced attention effect relays the switch and enables the registering of more mental events (Lejeune, 1998).

Notably, presenting emotional faces may induce a different subjective influence in viewers. Droit-Volet et al. (2004a) reported that presenting different emotional expressions influences the perception of time similarly. “Time dragging” resulted regardless of the emotional expression presented, though an angry expression induced the largest effect. A potential explanation is that the ecological significance of facial emotion among humans highly arouses the perceiver, speeding up the pulse generator, leading to an overestimation of the amount of time that has passed. Recent research has provided empirical evidence of this ecological significance (Doi and Shinohara, 2009; Kliegl et al., 2015), showing that the time-drag effect is maximal if an angry expression is presented directly to participants, whereas the effect diminishes when the gaze moves away. Such observations could be linked to the neuroanatomical pathway that enables preferential processing of affective stimuli in the amygdala (LeDoux, 2003). This thalamatic-amygdala “affective information highway,” proposed by LeDoux et al. (1999), serves the function of passing coarse visual information into the amygdala for quick evaluation (Morris et al., 1998; de Gelder et al., 2005). This “affective information highway” enables an organism to quickly identify potential threats in the environment, divert attention to a threat, and come up with an appropriate action plan, ultimately enhancing the chances of survival. This effect is very compatible with the attentional account, according to which emotion influences time perception, but empirical evaluation is scarce.

One limitation of previous studies is that the stimuli used are mostly static: static photographs and pictures (Angrilli et al., 1997; Droit-Volet et al., 2004a,b; Gil and Droit-Volet, 2011). A static facial expression is rarely seen in daily life, however; our facial expression changes all the time. If the perceived time-drag effect is due to the ecological significance of emotional expressions for humans, more attention will be drawn to process dynamically changing emotional expressions. This will lead to a larger overestimation effect. However, a dynamically changing emotional expression may also induce confusion over time. For example, when a face changes from a happy expression to an angry one, there are moments in between when no salient emotion can be perceived, which may reduce the overestimation effect. The latter hypothesis also implies that the perception of time is moderated by a real-time processing mechanism. Empirical testing is required to test how these two seemingly counteracting effects interact.

Further, it is known that different duration judgment paradigms affect perceived duration significantly (see Block et al., 2000 for a review). Knowing and looking forward to a time duration is different from recalling and reproducing a time duration. Similar characteristics may affect how emotions influence the perception of time duration. Lee et al. (2011) explored this with a two-interval time reproduction paradigm. Participants were asked to reproduce the duration of an emotional expression with a neutral stimulus (i.e., a gray oval) and reproduced the duration of a neutral stimulus with an emotional expression. The prior condition showed a similar time-drag effect (cf. duration bisection tasks), while the later showed a *time-fly effect*. Lee and colleagues' findings show that when emotional expressions were used as a relative reference, a similar time-drag effect was observed. Therefore, recalling and experiencing a static emotion expression appear to have a similar influence on time perception.

In the present study, we asked how stimuli with dynamic emotional information affect time perception. While previous studies have shown that a static expression exerts a uniform effect on time perception, it is possible that dynamic change of facial expressions affects us more than the emotion *per se*. For instance, if a neutral stimulus morphs into an emotion-inducing stimuli, is its effect greater or smaller than a static counterpart showing the emotion alone? If dynamic change matters, it is possible that changes in valence and arousal may result in different time perception. This unexplored setting of dynamic stimuli could provide novel information about the mechanism of emotional information processing. Specifically, the results will help us to test whether emotion affects time perception in real-time, prospectively, or retrospectively. If emotional information prospectively influences time perception via arousal effects, the emotional expression with which the morphing sequence starts will determine the strength of the time-drag effect. A retrospective influence account predicts that the morphing sequence of two emotional expressions (e.g., happy>angry vs. angry>happy) will lead to an identical time-drag effect because the total amount of attention captured by the morphing sequences will be the same. If the influence of emotion on time perception is a real-time process, dynamic emotional expressions starting with a neutral expression will present a lesser effect than dynamic emotional expressions starting with a positive or negative expression, because a positive or negative expression will capture attention and influence the internal clock processes immediately.

To confirm the role of emotion in time perception, the present study has adopted the methodology of Droit-Volet et al.'s study (2004a). We first tested the hypothesis that presenting facial expressions causes systematic error in time judgment. We also intended to investigate whether emotions' valence and arousal are related to a specific type of time distortion—a time-drag effect, a time-fly effect, or no effect—and how the change of valence and arousal may influence our perception of time. To achieve this, the present study selected three emotions, happy, sad, and angry, and a neutral facial expression in Experiment 1, and four emotions, happy, angry, afraid, and disgusted in Experiment 2. The present study also adopted a morphing technique in both experiments, for two purposes. First, since dynamic facial expressions have been found to enhance one's experience of emotional arousal (Sato and Yoshikawa, 2007), a morphing technique was adopted in Experiment 1 to investigate the effect of change in a particular emotion's arousal level on the type and accuracy of time perception. Second, since morphing techniques allow us to switch from one emotion to another smoothly and naturally, which in turn allows us to change both the level of valence and arousal, a morphing technique is adopted in Experiment 2 to assist in investigating the impact of change of an emotion's valence and arousal on time perception.

## Experiment 1 the influence of morphing emotional facial expressions on the perception of time

In Experiment 1, we examined how a dynamic emotional facial expression affects time perception. Perceived durations of static emotional expressions were also measured as a reference. We hypothesized that if emotion affects time perception in real-time, then a dynamic expression conveying a salient emotion for a shorter duration should exert less influence on time perception. If the influence is retrospective, on the other hand, a morph from a neutral to an emotional expression will induce an effect similar to static photographs.

### Stimuli

In this experiment, photographs of two models' emotional facial expressions, one male and one female, were chosen from a facial databank of nine models (five male and four female). The two chosen models were rated in the mid-range of the databank in terms of their emotional intensity (Female: 4.38; Male; 4.47; Mean: 4.45; *n* = 20) and attractiveness (Female: 3.36; Male: 3.86; Mean: 3.39; *n* = 36). We controlled the attractiveness ratings to minimize their potential influence on the perception of time due to induced pleasantness (Pruyn and Smidts, 1998). The intensity ratings indicated that the emotional expression of the chosen models were sufficiently intense to be identified. Four photographs of emotional facial expressions were chosen for each model: happy, angry, sad, and neutral. Thus, a total of eight photographs were used in Experiment 1.

**Figure 1:**
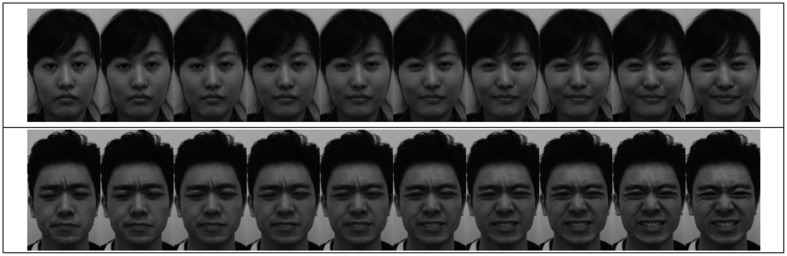
**Illustration of two sets of morphing sequence for a 400 ms stimulus**. The upper panel is a morph from a neutral to a happy expression of our female model (a morphing sample from Experiment 1). The lower panel is a morph from disgust to anger of our male model (a morphing sample from Experiment 2).

The photographs were morphed from the neutral expression to one of the three emotional expressions using MagicMorph provided by Etinysoft (http://www.effectmatrix.com/morphing/). Grayscale versions of the photographs were chosen to minimize any observable image blur during the morphing process. There were 50 frames in the morphing sequence; 48 frames were produced in between the two original images. The number of frames depended on the duration of the stimulus: each frame presented for 40 ms with DirectRT. For example, a 1000 ms stimulus presented 25 frames evenly distributed in the morphing sequence (i.e., all the odd number frames starting from 1st to 41st, then 44th, 46th, 48th, and 50th frames), each presented for 40 ms. Figure 1 shows two of the shortest sequences with 10 frames for a duration of 400 ms (1st, 6th, 12th, 18th, 23rd, 29th, 35th, 40th, 45th, and 50th frames). Sample stimuli are uploaded as Supplementary Materials.

### Participants

Forty-four undergraduates (31 female and 13 male) from Hong Kong Shue Yan University participated in this experiment. They received participation credits to fulfill the requirements of an introductory level psychology course offered by the Department of Counselling and Psychology. They were randomly assigned to different viewing conditions, which are described in the following Procedures section. Table 1 summarizes the participants' arrangement.

**Table 1:** **A summary of participants' arrangement in the experiments of the present study**.

	**Left-Long**	**Right-Long**
**Experiment 1 (***n* = 44**)**		
Female	8/3	8/4
Male	8/4	7/2
**Experiment 2 (***n* = 39**)**		
Female	8/2	6/4
Male	6/2	7/4

*Numbers in cells indicate the number of female/male participants. Rows indicate the viewing conditions*.

### Procedures

The procedures of the present experiment were similar to the setup of Droit-Volet et al. (2004a). Participants practiced the task viewing either a long presentation lasting 1600 ms or a short presentation of 400 ms on a computer screen (17” Topcon LCD). The stimuli in the practice trials were gray ovals subtending 3.7” × 3.8” on the computer screen. Participants had to judge the length of the presentations; their responses were collected by keyboard keys “D” and “K,” which were labeled “short” and “long.” The labels representing “short” or “long” were randomized across participants so that around half of them pressed “D” for “short and “K” for long (21 out of 44), and the other half did the opposite (23 out of 44). The first practice block comprised 10 trials of alternate 400–1600 ms presentations. Each trial was separated by a random inter-trial interval ranging from 1 to 3 s. A feedback screen with the correct answer was presented for 2 s after each participant's response. The procedure for the second practice was the same except that it consisted of eight trials of 400–1600 ms presentations shown randomly.

In the main experiment, the gray oval was replaced by a static face or a morphing sequence of expressions. They were presented for 400, 600, 800, 1000, 1200, 1400, or 1600 ms. The morphing stimuli thus comprised 10, 15, 20, 25, 30, 35, and 40 frames, each presented for 40 ms. Participants indicated whether they perceived the stimuli as closer to the long duration (1600 ms) or the short duration (400 ms) by pressing one of the two keyboard keys. Besides counterbalancing the left and right response, the gender of the model was also counterbalanced by the fact that around half of the participants were presented with the male model's photographs (21 out of 44) and the other half with the female model's photographs (23 out of 44).

The participants repeated nine experimental blocks and were allowed to rest freely in between. The whole experimental session lasted around 45 min. All experimental procedures were approved by the Human Research Ethics committee of Hong Kong Shue Yan University.

### Results and discussion

**Figure 2:**
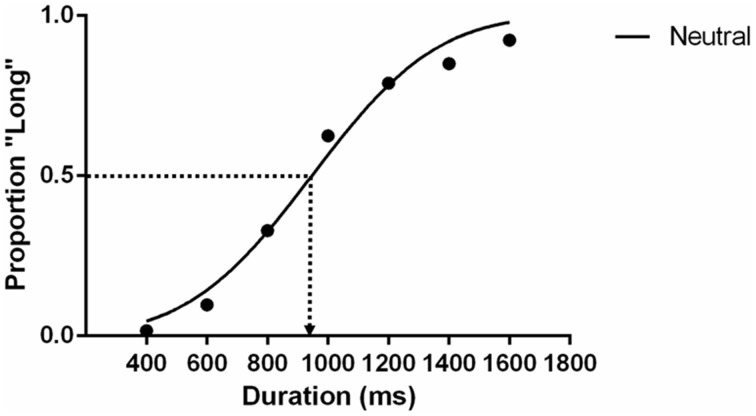
**The psychometric function of the static neutral expression condition in Experiment 1**. Each data point is the average proportion across all participants. The dotted line indicates the estimated PSE that participants give 50% of “long” responses.

There is no significant bias in female or male participants assigned to the two key press [χ^2^_(1, *n* = 44)_ = 0.38, *p* = 0.54] and stimuli gender condition [χ^2^_(1, *n* = 44)_ = 0.02, *p* = 0.89]. The potential key-press assignment and gender of the model were well-balanced, so they are collated and reported together in this section. Proportions of individuals' long-duration responses in every duration condition were computed. The data were then grouped according to emotional expression conditions. GraphPad Prism® 6 was used to analyse the data. Cumulative Gaussians were fit to the grouped proportion data using the maximum likelihood method. With this fitting of group data we estimated the Perceived Stimulus Equivalence (PSE), which indicate the stimulus durations participants were unable to identify as long or short. A graphical illustration of the PSE in the static neutral condition is shown in Figure 2. The estimated PSE (947.7 ms) is slightly shorter than 1000 ms. A PSE significantly shorter than 1000 ms implies overestimating perceived durations, equivalent to a time-drag effect. To test whether the differences are statistically significant, asymptotic standard errors and 95% confidence interval of the PSE estimates were approximated with the same statistical package. Although there is concern about the centrality of this approximation when the number of observations is small (number of intervals × trials in each < 150) (McKee et al., 1985), the sample of observations for the group fits in the present study is sufficiently large to satisfy this criterion (*n* = 2772). In order to further show whether PSE estimates conform to the normality assumption, we performed a Monte Carlo simulation by randomly flipping the participants' responses to create a null distribution for PSE. We obtained a null distribution for PSE from 10,000 permutations and the D'Agostino and Pearson omnibus normality test suggests that the distribution is normal (*p* = 0.79).

**Figure 3:**
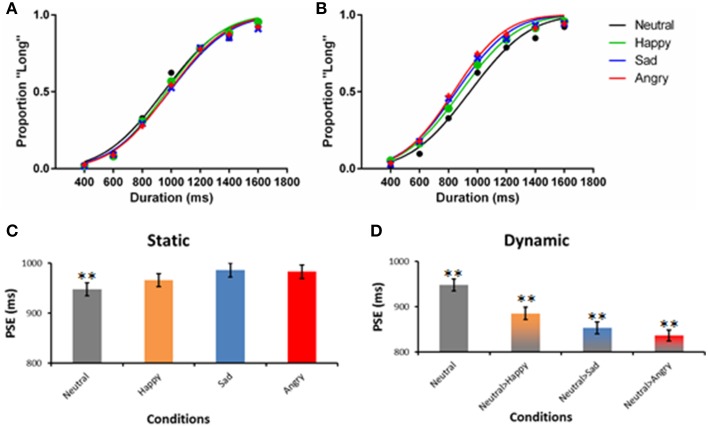
**Psychometric functions of Experiment 1**. The left panel **(A)** illustrates the fit of the static facial expression, whereas the right panel **(B)** illustrates the fit of the morphing expression. The fit of the static neutral expression is plotted in both panels as a reference. The lower panels **(C,D)** plot the same data using bar graphs, with the SEM of PSE presented. ^**^p < 0.01 in a one-sample *t*-test against 1000 ms.

Figure 3 shows the psychometric curves of the static (Figure 3A) and dynamic expressions (Figure 3B) in comparison to the neutral expression (*R*^2^ = 0.77–0.80, indicating good fit). Similarly, the 0.5 point on the y-axis illustrates the point where participants perceived the stimuli as being presented for 1000 ms. The PSE of static emotional expression mostly falls around 1000 ms. A subsequent parametric test shows that there is no significant deviation of perceived duration from the actual stimulus duration for all static emotion expressions. In contrast, the curves in Figure 3B have significantly shifted to the left, indicating an overestimation of duration; that is, a duration shorter than 1000 ms was perceived as 1000 ms, suggesting a time-drag effect. To better illustrate the results, the PSE of all seven conditions are plotted in Figures 2C,D together with their estimated standard error.

Among the four static facial expression conditions, neutral, and happy expressions are perceived to be significantly shorter than 1000 ms (Table 2). The PSE for sad and angry expressions were not significantly different from 1000 ms. The PSE of all three static emotional expressions were not significantly different from the neutral expression. The dynamic expressions, on the other hand, had significantly shorter PSE compared to their static counterparts [Happy: *t*_(86)_ = 4.23, *p* < 0.01, *d* = 0.91; Sad: *t*_(86)_ = 7.06, *p* < 0.01, *d* = 1.50; Angry: *t*_(86)_ = 8.06, *p* < 0.01, *d* = 1.72] indicating time-drag effects (see Table 2). Among the three dynamic conditions, the angry expression is the most overestimated.

**Table 2:** **Summary of one-sample *t*-test (test against 1000 ms) of the conditions tested in Experiment 1**.

**Expression**	**Estimated PSE (ms)**	***SE***	***t***	***df***	***p***	***d***
Neutral	947.7	13.41	3.90	43	< 0.01	0.59
Happy	965.8	13.19	2.59	43	0.01	0.39
Sad	986.3	13.70	1.00	43	0.32	0.15
Angry	982.9	13.66	1.25	43	0.22	0.19
Neutral>Happy	885.1	13.56	8.47	43	< 0.01	1.28
Neutral>Sad	853.1	12.99	11.31	43	< 0.01	1.70
Neutral>Angry	836.2	12.02	13.63	43	< 0.01	2.05

The dynamic facial expressions are found to induce time-drag consistently. The pattern of overestimation is similar to that reported by Droit-Volet et al. (2004a) and Tipples (2008), where the angry emotional expression was the most overestimated. Sad and happy were also overestimated but to a lesser extent. Their static counterparts, however, deviated less from 1000 ms and their deviations from the static neutral expression were not statistically significant.

There are three potential reasons why our static emotions do not induce substantial influence on participants' perception of time. An additional procedure in our setup differentiating it from the original study by Droit-Volet et al. (2004a) is the screening of attractiveness. The attractiveness of visual stimuli has been shown to affect the prompting of emotional states (Kenrick et al., 1993), which may in turn confound the effect of emotions on time perception. We chose two models from our photo bank who were not too distinctive but similar in attractiveness in order to minimize this potential confounder. Incidentally, the ratings of their emotional intensity also fall in the mid-range. The photographs chosen are perhaps not as salient as those in previous studies. Also, using black and white photographs may have further reduced the saliency of the emotions. The inclusion of a relatively large proportion of male participants in comparison to previous studies (Droit-Volet et al., 2004a; Tipples, 2008) may have further reduced the time-drag effect, which is more prominent in female subjects (Block et al., 2000).

Morphing facial expressions exaggerates the time-drag effect. This implies that the previously reported overestimation is not solely the result of a real-time mechanism. If the overestimation is an effect of real-time computation alone, morphing from a neutral expression to an emotional one should result in a diminished time-drag effect. In the current setup, given that all sequences started with a neutral expression, the participants had spent at least part of the stimulation time waiting for a readily perceivable emotional expression. Thus, the amount of time that participants were exposed to that emotion was reduced; the time in which it exerts an effect was in turn reduced. Our results imply that a pure real-time process of our internal clock cannot explain the results well. On the contrary, this process has to be retrospective: emotional expressions retrospectively expand the time-drag effect more than a static expression. A potential explanation is that a morphing expression resembles real life more than a static expression, thus magnifying the time-drag effect even though the emotion is only perceived about halfway into the stimulus' duration.

Another contributing factor could be the increased number of “events” experienced in a given time caused by the introduction of morphing. We argue that this cannot be the sole reason since the pattern of effects is highly reminiscent of those previously reported about static emotional expressions (Droit-Volet et al., 2004a; Tipples, 2008). If morphing is the sole reason for the magnification, the effect between emotions should be similar. Nevertheless, the dynamic conditions in this experiment provide references for comparison when morphing between two emotions is tested. Experiment 2 aimed to examine this in depth by testing the effect of morphing from one emotion to another.

## Experiment 2 the influence of morphing direction on the perception of time

The morphing sequences in Experiment 1 involved only one salient emotion. In Experiment 2, we examined how the perception of time is affected by a morphing sequence involving two emotional expressions. It is interesting to examine whether the two emotional expressions jointly influence the perception of time or hinder each other's influence such that the perceived duration should lie between the perceived durations of the first and second expressions. An alternative possibility is that participants' perception of time depends only on either the primacy or recency effect, that is, the beginning or the end emotion will determine the perceived duration. Nevertheless, there is also a possibility that the effect depends on the emotion changes involved only without any observable direction effect.

### Participants

Thirty-nine undergraduates participated in Experiment 2. Their arrangement in the experiment is shown in Table 1. All of them received participation credits for a psychology course.

### Stimuli

Four sets of emotional expressions were extracted from the two models chosen in Experiment 1. The emotional expressions represented happiness, anger, fear, and disgust. While a happy expression differs from the other three in terms of valence, angry, fear, and disgusted expressions differ from each other in terms of the level of arousal (anger>fear>disgust, see Figure 3). They were morphed in four sequences. The happy-angry pair allowed direct comparisons with the static and neutral>emotional conditions in Experiment 1. Fear was chosen to explore how approach-avoidance motivation influenced the perception of time as reported in previous literature (Marsh et al., 2005). Two pairs of fear-related sequences were prepared: happy-fear and angry-fear. To better understand the prominent effect of an anger expression, it was paired up with a disgust expression as well. All pairs were presented in both directions. Thus, there were eight morphing sequences of seven set durations (400, 600, 800, 1000, 1200, 1400, and 1600 ms).

### Procedures

The procedures were similar to Experiment 1. The first two blocks were a practice. Participants were randomly assigned to left-right and male-female viewing conditions (Table 1). They were allowed to rest in between blocks. The experimental sessions lasted less than an hour.

### Results and discussion

**Figure 4:**
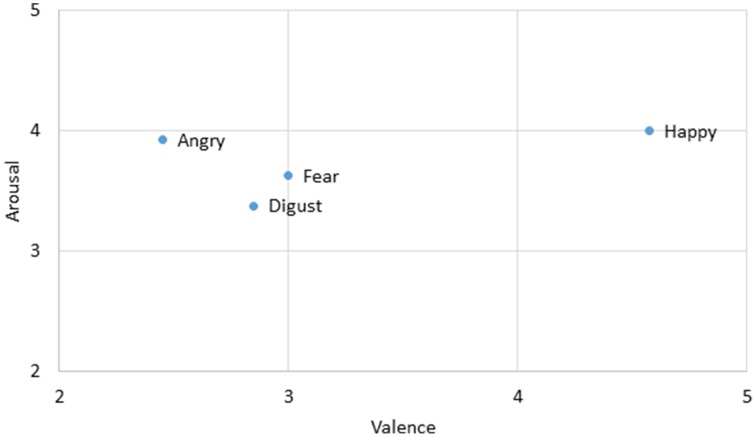
**Average valence and arousal ratings of the two models' photographs used in this condition on a seven-point scale rating task**. Data were obtained from a pilot study to rate the stimuli (*n* = 20).

The Chi-square statistics were similar to those in Experiment 1. Male and female participants assigned to the key press [χ^2^_(1, *n* = 39)_ = 0.24, *p* = 0.62] and gender of model conditions [χ^2^_(1, *n* = 39)_ = 0.01, *p* = 0.91] were unbiased. An additional Chi-square test was conducted to ensure that the proportions of participants' gender did not differ between Experiments 1 and 2 [χ^2^_(1, *n* = 83)_ = 0.01, *p* = 0.90]. Thus, the results of Experiment 1 provide a reasonable reference which is not influenced by gender difference. The key press and gender of model assignments were collated, and the proportions of “long” responses across time were fitted with cumulative Gaussian curves to estimate the PSE in each condition. Figure 4 summarizes the PSE of the conditions in Experiment 2 and their SEM. The first notable finding is a general time-drag effect in all conditions (Figure 4 and Table 3). In addition, all except the fear > angry and disgusted-angry pairs had significantly shorter PSE than the static neutral expression in Experiment 1 (Figure 4). This conforms to the findings in previous studies and our results in Experiment 1.

**Table 3:** **Summary of one-sample *t*-test (1000 ms) of the conditions tested in Experiment 2 (Static neutral expression condition is included for a comparison)**.

**Expression**	**Estimated PSE (ms)**	***SE***	***t***	***df***	***p***	***d***
Happy>Fear	836.3	14.66	11.17	38	< 0.01	1.79
Fear>Happy	820.3	12.11	14.84	38	< 0.01	2.38
Happy>Angry	874.9	13.42	9.32	38	< 0.01	1.49
Angry>Happy	863.7	13.14	10.37	38	< 0.01	1.66
Fear>Angry	922.9	14.59	5.28	38	< 0.01	0.85
Angry>Fear	884.7	16.57	6.96	38	< 0.01	1.11
Disgusted>Angry	941.5	15.85	3.69	38	< 0.01	0.59
Angry>Disgusted	892.4	14.59	7.38	38	< 0.01	1.18
Neutral (Experiment 1)	947.7	13.41	3.90	43	< 0.01	0.59

**Figure 5:**
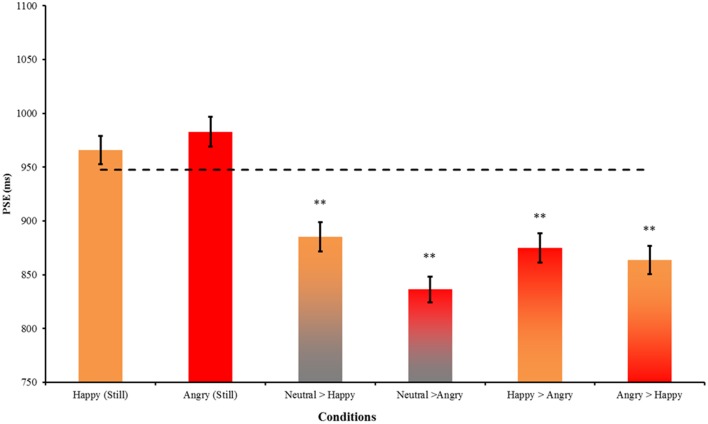
**A comparison of stimuli involving happy and angry expressions in Experiment 1 and Experiment 2**. Error bars indicate ± SE. The dotted line indicates the PSE of static neutral expression as the reference. ^*^^*^p < 0.01 in a *t*-test comparing the PSEs between this reference and the condition.

To further examine how dynamic changes between emotional facial expressions affect the perception of time, we regrouped the morphing pairs according to specific hypotheses concerning the induced approach-avoidance motivation, and valence and arousal changes. We first compared happy>angry morph with the neutral>happy and neutral>angry morphs in Experiment 1 (Figure 5). Dynamic expressions were found to have significantly stronger time-drag effects when compared to static expressions. In Experiment 1, the overestimation effect of the neutral>angry morph is significantly larger than the neutral>happy morph [*t*_(86)_ = 2.70, *p* < 0.01, *d* = 0.58]. Morphing between the happy>angry pair also induces a significant time drag, while the effect lies in between the two conditions. Both emotions appeared to exert an effect on the perception of time. This seems to indicate that a morph involving two distinct emotions has a time-drag effect that equals the average of the two neutral>emotional morphs. However, this interpretation remains a speculation and requires further testing because the happy>angry morphs are statistically not different from the neutral>happy or neutral>angry morph.

Among the eight pairs of dynamic emotional expressions, four pairs of morphing sequences with a happy face involve changes in valence whereas the rest involve mainly arousal changes (see Figure 3). To test whether the influence of valence change is greater or smaller than the influence of arousal change in the morphs, we regrouped the morph pairs accordingly. Additional *t*-tests comparing morph pairs involving happy and those without (the first four pairs against the last four pairs in Table 4) revealed a significant difference between these two groups [*t*_(6)_ = 3.381, *p* = 0.01, *d* = 2.76], suggesting that dynamic expressions involving major valence change are perceived as significantly longer than those involving arousal changes only. A change in valence in the morph sequence can potentially trigger the approach-avoidance motivation system, which in turn captures individual's attention, and as a result relays the switch of the internal clock for time perception.

**Table 4:** **Comparison of PSE across pairs of dynamic expressions of opposite directions**.

**Expression pairs**	**Mean difference (ms)**	***t***	***df***	***P***	***d***
Happy < > Fear	16.00	0.84	76	0.40	0.19
Happy < > Angry	11.20	0.60	76	0.55	0.14
Angry < > Fear	38.20	1.73	76	0.09	0.39
Angry < > Disgusted	49.10	2.28	76	0.03	0.52

Among the emotional facial expressions tested, an angry face was the most prominent trigger for the approach-avoidance motivation system. Therefore, examining the time-drag effect associated with the morphing sequences involving angry faces should give insight into this arousing, ecologically important stimulus. There is a significant difference between the two directions of the angry-disgusted pair [*t*_(76)_ = 2.28, *p* = 0.03, *d* = 0.52]. Similar trends of directional effects are observed in the other pairs involving an angry expression, though the differences are not statistically significant (see Table 4). Dynamic expressions starting with an angry expression were perceived as longer than dynamic expressions starting with other expressions and terminating with an angry expression. Such a pattern suggests that the arousing, avoidance-motivating angry expression has a primacy influence on the internal clock, which is consistent with the known neuroanatomical pathway between the thalamus and amygdala for quick processing of environmental cues of threats.

## Overall discussion

In the present study, we have examined whether dynamic facial expressions induce similar effects on humans' perception of time as static facial expressions. The effects are found to be similar and exaggerated for dynamic facial expressions. The time-drag effect persisted when morphing sequences involve two salient emotional expressions. A morphing sequence involving a valence change results in a larger time-drag effect than a morphing sequence involving arousal changes. Among all the pairs tested, those involving an angry expression demonstrated a primacy arousing effect in which angry-first sequences are perceived as longer than angry-last sequences. The implications of these findings are discussed below.

Experiment 1 explores the time-drag effect of dynamic expressions consisting of neutral>emotional pairs. The time-drag effects are much greater than when presenting a static emotional expression alone. This magnification could be explained by two factors. First, the morphing sequence introduced additional events to the time interval which perturbs the pacemaker and results in a perceived expansion of time by increasing the mental ticks. We argue that this is not the sole reason since the magnification is unequal between emotions and the pattern of difference across different emotional expressions conform to that reported in previous studies. We postulated that the ecological relevance of morphing stimuli may further exaggerate the overestimation: static faces are rarely seen in everyday life; real-life facial expressions involve dynamic changes in facial muscle configurations during talking, eating, and swallowing. A morphing sequence of facial expressions thus resembles a “real face” more than a static photograph, thus conveying richer emotional information and capturing more attention.

We further investigated the potential mechanistic influence of emotional facial expressions on the internal clock by using different morphing sequences in Experiment 2. Besides introducing dynamic changes as in Experiment 1, the morphing sequences in Experiment 2 involved two distinct emotional expressions. The results indicate that most pairs do not show any directional effect: there is little difference in the time-drag effect of the same pair of emotions regardless of their starting and ending emotions. The magnitude of the time-drag effect appears to fall between presenting the morphing sequence of the two expressions alone (i.e., neutral>emotional conditions in Experiment 1). If we assume that the time-drag effect is proportional to the exposure time for each emotional expression, a morphing sequence consisting of two emotional expressions should show a time-drag effect falling in between showing the two emotion expressions alone. Our finding seems to show such a trend. This interpretation, however, speculative since there is no statistical significant difference between the conditions and it also suffers from a confound: the frames in the middle of the morph sequence are ambiguous in nature. These frames do not convey salient emotional information; therefore their effects on time perception of a specific emotion are questionable. If emotional information influences time perception in a real-time manner, the reduction of salient information during these ambiguous frames would lead to a reduction of the time-drag effect, which is not support by the findings in the present study.

Among all the emotional expressions tested, the angry expression is the most prominent contributor to the time-drag effect. This conforms to studies in previous settings that revealed angry facial expressions as having the most significant effect on humans' perception of time (Droit-Volet et al., 2004a; Tipples, 2008). Another analysis indicated that an angry expression elicited a larger time-drag effect when presented first in a sequence than when presented at the end. It prospectively affects the subsequent experience of time perception. This could be due to the arousing effect of this prominent emotional expression on avoidance-related motivation (Marsh et al., 2005). This behavioral finding aligns with the neuroanatomical evidence for a preferential processing of potential threat information in the brain. Also, arousing the participants at the beginning of a sequence can trigger a larger time-drag effect, which provides evidence for a prospective effect of emotion on time perception.

**Figure 6:**
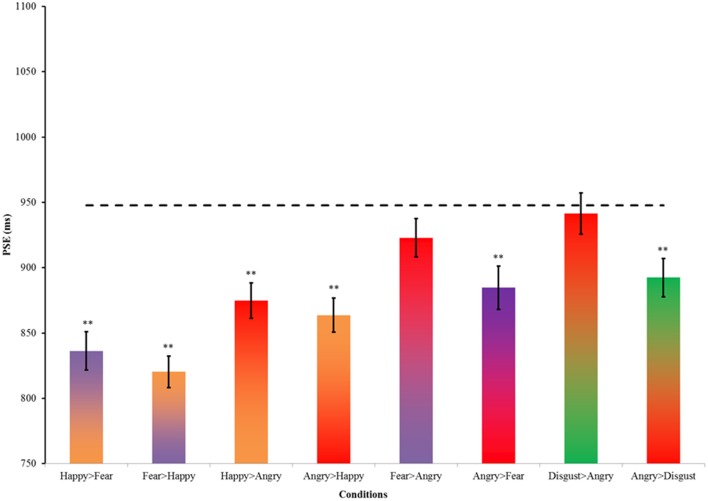
**Summary of PSEs in Experiment 2**. Error bars indicate ± SE. The dotted line indicates the PSE of static neutral expression as the reference. ^**^p < 0.01 in a *t*-test comparing the PSEs between this reference and the condition.

Dynamic expressions involving a happy expression also appear to amplify the time-drag effect. A contributing factor could be changes of valence in the dynamic expressions. Among all the pairs in Experiment 2, dynamic expressions involving a happy expression involve a significant valence change (Figure 6). Other pairs of angry, disgusted, and fear expressions involve mainly changes in arousal. A valence change in emotional facial expressions potentially provides more information than arousal change for signaling approach or avoidance related motivation. In contrast, change in the arousal of an individual affects the immediacy of a reaction. For example, both sad and angry expressions hint at an avoidance reaction, though the latter signals an immediate escape while the former only hints at a reconsideration or a change of approach. A recent study shows that stimuli signaling a voluntary go/no-go action induce significant time distortion (Yabe and Goodale, 2015). An evaluation of valence change requires a clear recognition to the emotion at both ends, which is only achievable retrospectively. Future neuroimaging studies may explore whether this effect can be found in the affective information pathway.

There is abundant research reporting the effect of emotion on the perception of time. Most reports a time-drag effect (Droit-Volet et al., 2004a) but there are also contradictory reports showing that presenting emotions at different times induces a reverse effect (Lui et al., 2011). These studies, however, exclusively adopted unified static emotional stimuli and have not explored the potential influence of dynamic emotional changes on the perception of time. The present study reveals a set of novel findings regarding the influence of emotional information on the perception of time. Dynamic expressions amplify the time-drag effects of emotions. As discussed, intermediate frames between the two ends of a morphing sequence can be ambiguous and do not convey salient emotional meaning. This may speed up the internal clock by introducing ambiguous events that capture participants' attention, but has little to do with the actual emotional meaning. Thus, a real-time process is unlikely to explain how emotion influences the perception of time. Amplification of the time-drag effect of angry-start sequences suggests a prospective influence of emotion on the perception of time. According to the internal clock model, emotional expressions, especially those conveying cues of potential threats, capture the viewer's attention and speed up the pacemaker. Nevertheless, emotion also appears to modulate the perception of time retrospectively. The complex meaning of stimuli—for example, changes in valence—also amplifies the time-drag effect, which can only be achieved retrospectively.

A notable limitation of our study is the artificial nature of our stimuli. Though, the morphing sequences we used provide better ecological validity than static photographs and pictures, they do not perfectly reproduce changes in real facial muscles. Nevertheless, given the similar pattern of our results to previous findings in various emotional conditions, the morphed frame should convey sufficient emotional information to participants. Besides, the morphing technique is limited in producing a dynamic expression without any emotion change. This condition will provide data in untangling the effects of emotion and pure facial movements in perceived duration. Morphing stimuli with perceivable facial changes requires two photographs differ significantly which usually entails changes in the perceived emotion expressions. In order to fully address these two limitations, real videos should be adopted (e.g., Botvinick et al., 2005) in future studies. This will help to understand the effect of real facial change on time perception. Also, models may perform action with their facial muscles without significant emotion expressions, for example chewing (Zhu et al., 2013; De Winter et al., 2015).

In summary, our results show that emotional information affects the perception of time at different time points. A retrospective process is evident across general emotional categories. A prospective process is also evident when motivations related to stimulus signaling approach or avoidance are involved. With the present setup, the evidence for a real-time effect on the internal clock appears to be weak. Despite the common belief that emotional processing is primitive and quick, its effect on time perception takes time. This delay diminishes its effect on time perception when emotional information changes in real time.

### Conflict of interest statement

The authors declare that the research was conducted in the absence of any commercial or financial relationships that could be construed as a potential conflict of interest.
